# Editorial: Genome Editing to Treat Cystic Fibrosis and Other Pulmonary Diseases

**DOI:** 10.3389/fgeed.2022.917916

**Published:** 2022-06-09

**Authors:** S. Vaidyanathan, A. L. Ryan

**Affiliations:** ^1^ Department of Pediatrics, Stanford University, Stanford, CA, United States; ^2^ Department of Anatomy and Cell Biology, Carver College of Medicine, University of Iowa, Iowa City, IA, United States

**Keywords:** gene editing (CRISPR/Cas9), cell therapy, engraftment, cystic fibrosis, basal cells, airway

**FIGURE 1:**
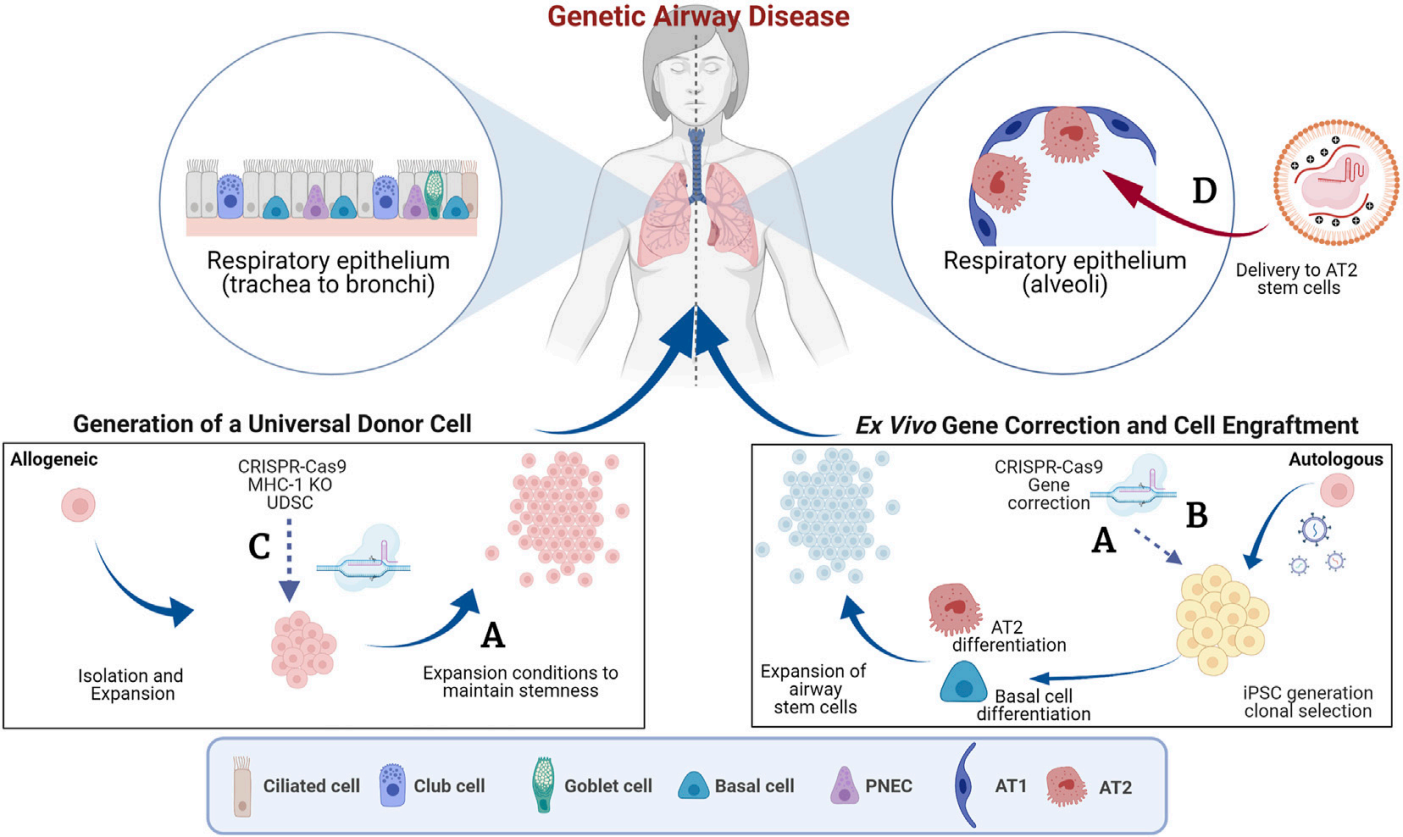
Genome editing to target the airway epithelium. This schematic summarizes the concepts features in the Research Topic. The blue arrows indicate pathways of *ex vivo* manipulation and subsequent delivery to the lung and the red arrows indicate direct delivery. **(A)** Generation of autologous gene-corrected airway basal cells (BCs) and expansion of primary BCs *ex vivo* (Barilla et al.). **(B)** Use of a double nicking CRISPR-Cas9n and intrachromosomal homologous recombination to overcome vector genome insertion events (Suzuki et al.). **(C)** Generation of beta-2 micro-globulin (B2M) knock-out (KO) cells to reduce immune rejection of cell therapies (Kararoudi et al.). **(D)** Delivery of gene editing tools to the distal airways using viral and non-viral approaches (Cooney et al.). AT1, Alveolar type 1 epithelial cell; AT2, Alveolar type 2 epithelial cell; iPSC, induced pluripotent stem cell; MHC-1, Major histocompatibility complex 1; PNES, pulmonary neuroendocrine cells. Created with BioRender.com.

The advent of genome editing tools, such as CRISPR-Cas9, has enabled the development of genetic and cell-based therapies for treating genetic diseases (Porteus, 2019). Multiple clinical trials are in progress to test the safety of autologous gene-edited hematopoietic stem cells (HSCs) to treat inherited blood disorders (NCT03655678, NCT04208529, NCT04853576, NCT04925206, NCT04819841, NCT04774536) and *in vivo* genome editing of the liver to treat Transthyretin amyloidosis (ATTR, NCT04601051) or Hereditary Angioedema (HAE, NCT05120830) (Frangoul et al., 2021; Gillmore et al., 2021). Notably, most of the open clinical trials currently focus on gene Knock-out (KO) rather than homology directed repair of genes. KO of gene function does not require the simultaneous delivery of a homologous sequence to correct a disease-causing mutation and, therefore, is usually associated with higher efficiencies of successful editing. The feasibility of these examples was accelerated due to our already extensive knowledge and established procedures for HSC transplantation in the bone marrow (Consiglieri et al., 2022) and availability of lipid nanoparticle technology that efficiently targets the liver (Qiu et al., 2021). Unfortunately, such techniques and technologies are not available for targeting the lung specifically, therefore, expanding the use of genome editing tools to treat other inherited disorders, such as cystic fibrosis (CF), primary ciliary dyskinesia (PCD) and surfactant protein disorders impacting the lungs is of significant interest. In these instances, *in vivo* genome editing is limited by challenges in 1) delivery of the genome editing reagents to the desired cells, homologous recombination needed for gene correction requires CRISPR-Cas9 and homologous DNA to be delivered into the same cell and 2) understanding of the ideal cell/stem cell to target for long-term disease correction. *Ex-vivo* gene editing is likely a more efficient approach, however delivery of gene-edited cells and conditioning regimens that make the epithelium receptive to the engraftment of the cells without compromising lung function in patients still present significant challenges. In this Research Topic, we feature four articles which describe efforts to produce autologous gene-corrected airway basal cells (BCs), transplant airway BCs and discuss the potential to expand these tools to treat surfactant protein disorders that affect the alveoli. Figure 1 summarizes the findings of these studies.

One major challenge is the efficient gene correction of airway stem cells while maintaining their regenerative potential. Many gene correction efforts have focused on CF as it is one of the most well-characterized genetic disorders affecting the lungs (Suzuki et al., 2020; Vaidyanathan et al., 2020). CF is caused by mutations in the CF transmembrane conductance regulator (CFTR) gene. Over 2000 different mutations have been described in CFTR and as a result there has been tremendous interest in replacing the entire CFTR coding sequence to develop a therapy applicable to all CF patients. However, the CFTR coding sequence (4,500 bp) is close to the packaging limit of commonly used adeno-associated virus (AAV) vectors (4,800 bp) making this challenging. In this Research Topic, Barilla et al. describe the tremendous progress that has been made in inserting the partial and full-length CFTR cDNA in the endogenous CFTR locus in induced pluripotent stem cells (iPSCs) and airway BCs. Aside from challenges in efficient gene correction, there have been concerns that the significant expansion of airway BCs *in vitro* limits their ability to form epithelia with functional chloride transportation via the CFTR (Suzuki et al., 2020). The authors present promising data on the use of modified culture conditions to overcome this reduction in the regenerative potential of airway BCs.

Safety concerns surrounding genome editing add additional apprehension on moving this approach into patients (Blattner et al., 2020). Genotoxicity is always a concern when carrying out a process that may impact DNA integrity or damaging DNA. Suzuki et al. describe the correction of a single causative mutation in CF, W1282X, in iPSCs followed by clonal selection using puromycin resistance. They report that vector genome insertion events created partial duplications in the targeted exon 23 of CFTR in the corrected clones and that this could be overcome by intrachromosomal homologous recombination using a double nicking approach with CRISPR-Cas9n. Although the subsequent development of highly efficient selection-free genome editing has limited the need for clonal selection, the study highlights the importance of thorough characterization of the genomic integrity of gene-corrected cell-based therapies.

The advances in genome-editing approaches have stimulated considerable interest in strategies to transplant engineered airway BCs. Kararoudi et al. report the use of CRISPR/Cas9 to knockout rat Beta-2 microglobulin (B2M) in tracheal BCs to create a universal donor stem cell (UDSC). Knockdown of B2M, a component of the Major Histocompatibility Complex (MHC-I), should reduce the chance of rejection by the recipient’s immune system, enhancing the chance for long-term engraftment. The ability of the UDSC to treat lung injury caused by the inhalation of sulfur mustard, a chemical warfare agent was evaluated, and the study demonstrated that these cells retained an ability to self-renew and undergo multilineage differentiation. While promising, it is now critical to follow on from these studies and develop more clinically applicable conditioning regimens and larger, more-relevant, pre-clinical animal models to facilitate the transition to transplantation of airway BCs in humans.

*In vivo* targeting of more distal airway cells, namely the alveolar type 2 (AT2) epithelial stem cells, is also of substantial interest to the field of airway and lung regeneration. Surfactant protein disorders driven by pathogenic mutations in genes specific to AT2 cells including pulmonary surfactant proteins B and C (SFTPB, SFTPC) and ATP-Binding Cassette transporter A3 (ABCA3). The featured mini review by Cooney et al. describes genes associated with surfactant protein disorders and the progress, to date, in advances toward genetic therapies for these diseases. This mini review describes the efforts that have focused on the direct replacement of missing genes using viral (AAV) or non-viral vectors or *in vivo* genome editing (Stribling et al., 1992; Tu et al., 2000). Major challenges that remain to be resolved pertain to specific design of the viral vectors, their tropism of the target cell type and, as for the other studies, appropriate pre-clinical models to evaluate function. Specifically, for many lung diseases the efficacy of transducing cells, promotor activity and packaging limitations of AAV are considerations to be considered when designing such vectors.

Overall, these studies highlight the promise that genome editing holds to treat CF and other genetic airway diseases. While substantial progress has been made in the development of methodology and technology to specifically target the human lung there are still many challenges to overcome. Editing approaches to insert the full-length and partial CFTR cDNA in airway BCs has now paved the way for efforts to optimize BC expansion *ex vivo* and transplantation. AAV, lentiviral, and adenoviral (Ad)-based vectors as delivery vehicles are being used to develop gene addition and gene editing strategies to efficiently target the respiratory epithelium and UDSC are being created to potentially reduce immuno-rejection of cellular therapies and enhance successful long-term engraftment. As the technologies continue to develop, adapting then to generate targeted cellular therapeutics for treating genetic diseases of both the proximal and distal airways will continue to be a focus of the respiratory field.
